# 

**Published:** 1920-02-28

**Authors:** 


### TUBERCULOSIS NOTES.

### Brompton Hospital Appeal.

The Brompton Hospital for Consumption, now seventy-seven years old, is seeking to build an extension to meet the needs of those discharged from the Navy and Army and prison camps suffering with tuberculosis. Lord Cheylesmore, the chairman, states that £50,000 will be requisite. The report of the Inter-Departmental Committee, which we have noticed previously, has made it clear that a serious deficit exists in the matter of sanatorium beds for males, of which at present there are only some 19,000 in the country. It is, therefore, to be hoped that from one source or another Lord Cheylesmore's appeal will receive substantial support.

### Chemo-Therapy.

Schroder, of the Tchömberg Sanatorium, discusses the application in the case of consumptives of the newer chemo-therapy of tuberculosis. Copper preparations in lung tubercle he rejects as useless, and even dangerous. But gold preparations are useful intravenously, and seem to have a specially good effect upon laryngeal tuberculosis. "Auro-cantan" may be employed in doses of one-third to one gram. A preparation five times less toxic, however, is the sodium salt of a very complicated base—amino-auro-phenol-carbonic acid, to which the name of Krysolgan has been given. The great Höchst factory issues this. Injection of this produced slight focal reaction in the tuberculous larynx, in the form of redness, slight swelling, and increase of secretion; the dose is recommended to be repeated at ten-day intervals. "Gold therapy" should be combined with all other anti-tuberculosis therapeutic means.

### Consumption "Cures."

Often do lay newspapers, even leading ones, announce the appearance of meteoric certain cures for consumption, but seldom do they chronicle their utter extinction. Our contemporary *Truth* forms a praiseworthy exception. It records a disillusioning trial of the Italian sugar cure. But some months ago an Italian professor wrote to the *Lancet* in just the same sense. Really there is a great need of a supplement to the famous "Secret Remedies" volume, dealing exclusively with consumption cures, especially quack ones. Where is now the "maggot cure" extolled by Mr. John Burns, or many a dozen other nostrums? The fallacies swallowed by lay (and indeed medical) judgment are painfully shown in the history of phthisio-therapeutics.

### Cheshire Colony for Consumptives.

The Cheshire County Council have lately had before them a munificent offer from the British Red Cross Society to give a grant of £20,000 towards the establishment of a working colony for the care and employment of cured and arrested consumptives. Subject to the Local Government Board (or as one should say now the Ministry of Health) fulfilling its usual agreement to provide three-fifths of the capital expenditure and one-half the annual cost of maintenance of such an institution, it was decided to accept the offer and to delegate to the Public Health Committee the dealing with the matter, the chairman remarking that they could not put the £20,000 to a better purpose. It was proposed to apply this sum to purchasing the property for the colony, which is to have accommodation for fifty men, with facilities for expansion, and to acquire a modern country house with from fifty to a hundred acres of land, an area which in such a rich agricultural district may prove adequate.

### No Milk for Babies.

That the high price of milk is having a bad effect on the nutrition of babies is proved by a report which the Medical Officer of Health has submitted to the Glasgow Corporation. He quotes from nurses' reports, which indicate that poor mothers are forced to give their infants tea with toast soaked in it, and that porridge is disappearing in some districts owing to the high price of milk and oatmeal. As the Medical Officer says, the result will inevitably produce a generation whose natural resistance to disease will be so lowered that ill-health and increased susceptibility to infectious disease will result.

### PASSING EVENTS AND TOPICS.

### The Hospital Problem in Cardiff.

No district in the whole of the country was so badly served with hospital accommodation as South Wales, said Mr. Leonard D. Rea, superintendent of the King Edward VII. Hospital, Cardiff, at the weekly luncheon of the Cardiff Rotary Club. Mr. Rea spoke of the housing problem as it affected the hospital, and remarked that no question confronting the matron at the present time was fraught with greater difficulty. To it could be traced the root of the industrial unrest, but hospitals had always been faced with the problem. The great programme of the years from 1901 to 1908 ended by the erection of the Colonel Bruce Vaughan wing, when the hospital was brought up to its present accommodation of 272 beds. South Wales was, however, far behind other districts. Whereas a patient could gain admission to Bristol Hospital within a week, a matter of six months might pass before he could enter the hospital at Cardiff. This was attributable to the rapid growth of the district. Now at the King Edward VII. Hospital they had almost completed the extensions started in 1916. When the extensions were completed accommodation would be provided for 453 beds, sufficient to deal with 6,342 patients yearly. Had this scheme been deferred for four or five years, instead of costing £100,000, it would have amounted to £250,000.

### A South African Protest.

The Chairman and four other members of the Boksburg Hospital Board resigned at a meeting held on December 22 on account of alleged discourtesy to the Board shown by the Transvaal Provincial Administration. The Chairman said the members of the Board had been treated like office boys, and he would at once notify their resignations to the Provincial Secretary. The action of the Provincial Administration which has given offence to the Board is the publication in the *Provincial Gazette* of the following notice: "Nurses, when taking their weekly day or night off, as the case may be, in accordance with the provisions of the Hospital Nurses' Relief Ordinance, 1917, may be absent from the hospital premises for the night immediately preceding their day off, and night nurses for the night on which they are not on duty; provided that before leaving the hospital premises they enter in a book, to be kept for the purpose, the addresses at which they can be found during the night." The Chairman (Mr. Fraser) said that this regulation struck at the root of hospital management and discipline. The Board, by a majority, the matron, and twenty-two out of twenty-three parents, were against the regulation, and the fact had been made known to the Provincial Executive. One member only of the Board supported the action of the Provincial Administration—Mr. G. Hills, M.P.C.

### Children's Hospital for the Rand.

The provision in the Johannesburg General Hospital for the needs of children is very inadequate, only forty-four beds being set aside for them. A public movement has been launched for the building of a children's hospital capable of taking in a hundred patients, and situated, for convenience of administration, near the General Hospital. A sum of £45,000 will be required for the work.

### Appeal for £50,000.

The Alexandra Hospital for Hip Disease appeals for £50,000 to enable the Committee to accept the offer of the Red Cross of £25,000 towards the scheme for removal to Caversham, where part of the new buildings, it is proposed, will be used for tuberculous ex-Service men.

### The League of Mercy (Wanderer's Branch).

The Council of the League of Mercy recently sanctioned the formation of a Wanderers' Branch, which seeks to organise for the benefit of the hospitals all who have wandered, or are still wandering, round the world. Vice-Presidents subscribe £1 1s. and enlist members. Members endeavour to collect not less than £1 annually; associates subscribe 1s.; honorary members subscribe a guinea a year, but undertake no duty. Readers who are interested should write to the President of the Wanderers, Colonel R. J. Blackburn, C.B., D.S.O., at the headquarters of the League of Mercy, 29 Southampton Street, Strand, W.C.

### St. Mark's Hospital.

The Lord Mayor, presiding on February 12 at the eighty-fourth annual meeting of St. Mark's Hospital for Cancer, Fistula, and Other Diseases of the Rectum, City Road, E.C., drew attention to the fact that £2,300 is owing to the bankers, and appealed for help to wipe out that debt and to meet ordinary expenditure. During the year the hospital treated 1,509 out-patients and 655 in-patients. That the latter number was lower than in 1918 was stated to be partly due to an increase in serious cancer cases requiring long, painstaking treatment.

### Memorial to a Mayor.

The January meeting of the board of the Leicester Royal Infirmary was noteworthy from the fact that a relative of the late Mr. William Barfoot, Mayor of Leicester in 1875-76, was unexpectedly in attendance to ask the board's acceptance of a cheque for £1,000 for the endowment of a bed. The donor was a daughter-in-law who desired to perpetuate his memory in the city by endowing a bed as a memorial. Mr. T. Fielding Johnson, junr. (chairman), accepted the gift with gratitude and referred to the pleasure it gave the board that the infirmary had been selected as a means of perpetuating the work, name, and memory of a former mayor and worthy citizen.

### The Staff at Bermondsey Hospital.

Bermondsey Board of Guardians are filling up the vacant positions at their infirmary at Rotherhithe. At the last meeting Dr. H. P. Ashe, M.R.C.S. (Eng.), L.R.C.P. (Lond.), was appointed first assistant medical officer at a salary of £350 a year, rising by annual increments of £25 to £450 He is at present at King's College Hospital, and was a captain in the R.A.M.C. Dr. Robert Welch, M.B., B.S., B.Sc., at present an assistant medical officer at Mile End Infirmary, was appointed second assistant medical officer at a salary of £300 a year. The Guardians have been informed by Dr. J. T. Macnamara, formerly medical officer at the Ladywell Institution, that his commission in the R.A.M.C. will terminate on March 11. The institution is now controlled by the War Office, and until it is handed over to the Guardians Dr. Macnamara has informed them that he places his professional services at their disposal in any suitable capacity.

### Medical Appointments.

MR. RUSSELL J. HOWARD, M.S., F.R.C.S. (senior surgeon), and Mr. C. Jennings Marshall, M.D., M.S., F.R.C.S., have been appointed to the hon. visiting staff of Manor House Hospital, Hampstead.

DR. GEORGE GRAHAM and Dr. W. J. Adey have been appointed physicians (with charge of out-patients) at the Great Northern Central Hospital, Holloway, and Mr. M. J. K. Souter, L.D.S., R.C.S., has been appointed assistant to the dental surgeon.

### Matrons' and Sisters' Appointments.

BOROUGH TUBERCULOSIS HOSPITAL, NORTHAMPTON.—Miss Amy Burras and Miss M. Cardwell, A.R.R.C., have been appointed sisters. Miss Burras was trained at the Cumberland Infirmary, Carlisle, and at the Plaistow Fever Hospital, has been staff nurse at the Royal Victoria Infirmary, Newcastle-on-Tyne, and sister, night superintendent, and home sister at Baguley Sanatorium, Cheshire, and has also done private nursing. Miss Cardwell was trained at the Prince of Wales General Hospital, London, has been sister at Mount Vernon Hospital, Northwood, night sister at the Manton Grange Hospital, Rutland, ward sister at the Edmonton Military Hospital, and matron of the Southgate Auxiliary Hospital, Palmer's Green. Miss Cardwell has also done district and private nursing.

ISOLATION HOSPITAL, SKIPTON.—Miss M. Willcocks has been appointed sister. She was trained at West Ham Union Infirmary, and has been staff nurse at Plaistow Fever Hospital and at the Whipps Cross War Hospital.

ISOLATION HOSPITAL, NORWICH.—Miss Mary McKinnon has been appointed night sister. She was trained at the General Hospital, Rochdale, and the City Hospital, Aberdeen, at which latter institution she was later charge nurse. Miss McKinnon has also been ward sister and home sister at the City Hospital, Little Bromwich, Birmingham, has served under Queen Alexandra's Imperial Military Nursing Service Reserve both in England and France, and has been mentioned in despatches.

EVESHAM UNION INFIRMARY.—Miss Lilian Maud Stephens has been appointed head nurse. She was trained at Hunslet Union Infirmary, Rothwell, Leeds.

TAMWORTH HOSPITAL, STAFFS.—Miss Annie G. Hughes has been appointed matron. She was trained at the Royal Infirmary, Oldham, has been sister at the Rochdale Infirmary, and at the Royal Eye Hospital, Manchester, and assistant matron of the District Hospital, Watford.

FALMOUTH HOSPITAL, CORNWALL.—Miss Marjorie Cox has been appointed sister. She was trained at the East Sussex Hospital, Hastings, has been staff nurse at the Wellington and District Hospital, Somerset, and sister in joint war hospitals at home and abroad.

ROYAL INFIRMARY, PERTH.—Miss Agnes Gourlay and Miss Agnes Shepherd have been appointed sisters. Miss Gourlay was trained at the Dumfries and Galloway Royal Infirmary, Dumfries, and at the Fever Hospital, Paisley, has been staff nurse at the Leith Public Health Hospital, Edinburgh, and sister at the Fever Hospital, Paisley. Miss Shepherd was trained at the Royal Infirmary, Hull, and has been sister at the Lincoln County Hospital. Both Miss Gourlay and Miss Shepherd were sisters in the Territorial Force Nursing Service, Miss Shepherd serving at home, and Miss Gourlay at home and abroad.

### No. 24 (The Welsh) General Hospital and tho Welsh Hospital, Netley.

A REUNION dinner is proposed in London towards the end of April for all ranks (men and women) of the above hospitals. Those wishing to attend should notify R. L. Mackenzie Wallis, Esq., 55 Townshend Road, N.W. 8, or E. J. August, Esq., 51 Etchingham Park Road, N. 3, before March 12.

### The College of Nursing (Limited by Guarantee).

OXFORD CENTRE.

THE second Executive Meeting was held on January 29. It was decided to try and form a Financial Committee, also to arrange lectures monthly (the first to take place on February 18), and to start a Club as soon as financially possible.

LIVERPOOL CENTRE.

MARCH 17, 7 P.M.—In the Lecture Theatre of the Liverpool Royal Infirmary, at 7 P.M., when Miss M. H. F. Ivens, M.B., M.S., will lecture on "What we have Learned in the War."

Miss Eleanor F. Rathbone's lecture, held this month on "The Civic Privileges and Duties of the Women Citizen," was keenly appreciated by the members of the Centre and others.

"THE BULLETIN."

THE College of Nursing has made a fine start with its new *Bulletin*, issued free to members as far as the first four numbers go, and at a charge of 6d. to the public. Its general appearance reflects credit on the printers and publishers. Members should cherish this first copy, which has a definite historic value. The contents are of importance to all members, and the matter is well put together. The synopsis of the Nurses' Registration Acts in England, Scotland, and Ireland is well arranged, and very useful for reference. But the chief interest of the number lies in the record of work accomplished in the twenty-three Local Centres of the College. It seems scarcely credible that none of these Centres were in existence before 1918, so much have they already accomplished, and so vast the latent energies they have set in motion. We offer our hearty congratulations to all concerned in this interesting College development.

### St. Thomas's Hospital House Appointments.

THE following have been selected as house officers from February 3, 1920: *Casualty Officers and Resident Anæsthetists*: J. D. M. Cardwell, M.R.C.S., L.R.C.P. (Ext.); A. F. Potter, M.R.C.S., L.R.C.P. (Ext.); H. G. Stormer, M.R.C.S., L.R.C.P. (Ext.); C. M. Billington, B.A.Cantab., M.R.C.S., L.R.C.P.; R. C. Cooke, D.S.O., M.C., M.R.C.S., L.R.C.P.; J. Hale, M.A.Cantab., M.R.C.P., L.R.C.P.; D. B. Spence, M.R.C.S., L.R.C.P.; F. G. Wood, M.A.Cantab., M.R.C.S., L.R.C.P *Resident House Physicians*: W. S. Dawson, M.A., M.B., B.Ch.Oxon., M.R.C.S., L.R.C.P.; R. C. P. Whitcombe, B.A.Cantab., M.R.C.S., L.R.C.P. (Ext.); S. A. T. Ware, M.R.C.S., L.R.C.P.; R. A. W. Procter, M.C., M.A.Cantab., M.R.C.S., L.R.C.P. (Ext.). *Resident House Physician (for Children)*: J. Forest Smith, M.R.C.S., L.R.C.P.. *Resident House Surgeons*: R. H. O. B. Robinson, B.A., M.B., B.C.Cantab., M.R.C.S., L.R.C.P.; J. W. Wayte, M.C., M.B., B.S.Lond., M.R.C.S., L.R.C.P. (Ext.); G. Perkins, M.C., M.A., M.B., B.Ch.Oxon., M.R.C.S., L.R.C.P.; R. M. Humphreys, B.A., M.B., B.Ch.Oxon. *Resident House Surgeon (for Ear, Nose, and Throat)*: H. I. Mariner, M.R.C.S., L.R.C.P. *House Surgeon to Block* 8: L. G. Higgins, M.A.Cantab., M.R.C.S., L.R.C.P. *Obstetric House Physicians*: A. H. Richardson, M.A., M.B., B.C.Cantab., F.R.C.S.Eng.; H. L. Garson, M.C., B.A.Cantab., M.R.C.S., L.R.C.P. *Ophthalmic House Surgeons*: J. P. S. Walker, M.A., M.B., B.Ch.Oxon.; E. P. Brockman, B.A.Cantab., M.R.C.S., L.R.C.P. *Clinical Assistants*: (Throat), J. B. Cavenagh, M.C., B.A., M.B., B.Ch.Oxon., M.R.C.S., L.R.C.P.; A. R. C. Doorly, M.B., B.S.Lond, M.R.C.S., L.R.C.P. (Skin), H. Gardiner-Hill, M.B.E., B.A., M.B.Cantab., M.R.C.S., L.R.C.P.; A. C. Halliwell, B.A.Cantab., M.R.C.S., L.R.C.P. (Ear), P. C. Brett, M.R.C.S., L.R.C.P.; N. A. Sprott, B.A., M.B., B.Ch.Oxon., M.R.C.S., L.R.C.P. (Electrical and *x*-ray department), F. G. Spear, B.A.Cantab., M.R.C.S., L.R.C.P.

### "DAILY TELEGRAPH" SHILLING FUND.

### NURSES' CONTRIBUTIONS.—Second Instalment.

**The following contributions to the Shilling Fund for Nurses have been announced in the "Daily Telegraph" of February 19 to 25.:—**

Shillings.

Irish Branch of the College of Nursing, for Endowment Fund: Mater Infirmorum Hospital, Belfast, 330s.; Royal Victoria Hospital, Belfast, 85s.; Royal City of Dublin Hospital, 18s.; some Dublin College Members, 67s. ... ... ... 500 0

College of Nursing, London Centre, second instalment, 170s.; Matron and Staff, King's College Hospital, 240s.; Matron and Staff, Whipp's Cross Infirmary, 25s. ... ... ... ... ... ... 435 0

Nursing Staff, Guy's Hospital ... ... ... 250 0

Per Nursing Staff, Royal Infirmary, Sheffield ... 244 0

Dundee Centre College of Nursing: Royal Infirmary, Dundee, 118s. 6d.; King's Cross Hospital, Dundee, 8s.; Miss Little's Private Nursing Staff and Home, 49s. 9d.; Miss Chapman's Private Nursing Home, 20s.; County Hospital, Forfar, 10s. ... ... ... ... ... ... ... 244 3

Nurses, Bristol Royal Infirmary ... ... ... 229 0

Staff of General Infirmary, Burton-on-Trent ... 200 0

Nursing Staff, University College Hospital ... 200 0

Lake Hospital, Ashton-under-Lyne, second instalment, per Miss Seymour Yapp ... ... ... 120 0

The College of Nursing, Ltd., Derby Centre: Royal Derbyshire Infirmary, 62s.; Isolation Hospital, Derby, 30s.; Royal Nursing Institution, 13s. ... ... ... ... ... ... ... ... ... 105 0

Ethel Scrymgeour, ex-Nurse ... ... ... ... 100 0

Hornby Division, British Red Cross Society ... 100 0

Members of the Glasgow Centre, College of Nursing ... ... ... ... ... ... ... ... 100 0

**Sums under 100s.:**

Marylebone Infirmary, N. Kensington, 89s.; Lincoln Centre College of Nursing (second instalment), 63s.; To Endow College of Nursing, from St. John's Hospital, Lewisham, 60s.; Matron, Swansea General and Eye Hospital, 60s.; Members of College of Nursing, Stockton and Thornaby Hospital, Stockton-on-Tees (Whist-drive), 60s.; Per Nurse Crosbie (second instalment), 59s.; Private Nurses of Westminster Training School, 52s.; The Nurses, Birmingham Union, 50s.; Per Royal Berkshire Hospital, Reading: No. 1 War Hospital, per Miss Steen, 16s.; Miss Hamilton's Nursing Home, 10s.; Nursing Staff, Royal Berkshire Hospital, 24s.—50s.; Staff of Royal Free Hospital (further donation), 43s.; Nurses, Addenbrooke's Hospital, 42s.; Dundee Centre College of Nursing (further subscription): Dudhope House, per Miss Winstanley, 22s.; Caird Home District Nurses, per Miss Milne, 8s.; Brechin Infirmary, 7s.; King's Cross Hospital (further subscription), 2s.—39s.; Staff of the Imperial Nursing Home, Cheltenham, 37s. 6d.; College of Nursing, Plymouth and District Centre (additional contribution), 35s.; St. Bartholomew's Hospital, Rochester (Staff), 34s. 6d.; Queen Victoria's Jubilee Institute, further contribution, 28s. 6d.; Nurses, Victoria Memorial Cottage Hospital, 26s.; Staff of East Lancs. Monsall Hospital, 25s. ... ... 853 6

**Sums of 21s. and under:**

Nursing Staff, Robroyston Hospital, 21s.; Matron, Victoria Hospital, Guernsey (College of Nursing), 21s.; "A Former London Hospital Sister," 21s.; Nurses, Royal Orthopædic Hospital, 20s.; Nursing Staff, Withington Hospital, 20s.; Sister Elizabeth, Isle of Wight, 20s.; Per Queen Victoria's Institute for Nurses, 18s.; G. Gellens, Queen's Nurse, Aberdeen, 16s.; Cornwall Centre, College of Nursing (second donation), 15s.; Staff, Ear and Throat Hospital, Birmingham, 14s.; Cornwall Centre, College of Nursing, Ltd., Truro (third instalment); Matron, Hospital, Liskeard, 5s.; Collected by Sister Bing, 9s.—14s.; Reading-ton Orthopædic Hospital, Oxford, 12s.; Exeter Trained Nurses Corporation, first instalment, 10s. 6d. ... 222 6

**Sums of 10s. and under:**

Some of the Nursing Staff, Gravesend Hospital, 10s.; E. Lancashire Collection, Grangethorpe Hospital, 10s.; "A Member," Birmingham Centre, 10s.; Matron, County Hospital, York (College of Nursing), 10s.; Matron, Gillingham Hospital, 10s.; Per Nurse Cotton Smith, Coventry and Warwickshire Hospital, 8s. ... ... 58 0

**Sums of 5s. and under:**

A Nurse trained twenty-eight years ago, now crippled, 5s.; I. Cliff, Leicester Centre, College of Nursing, 5s.; Nurse M. Barrie Scott, 5s.; Three Members of College of Nursing, Birmingham, 3s.; Durham Centre, College of Nursing, 3s.; Nurse Sleigh, 3s.; Hospital Sister, 2s. 6d.; From a Highbury V.A.D., 2s. 6d.; Nurse J. S. Martin, 2s.; Nurse C., 2s.; A Private Nurse, Windermere, 2s.; Nurses Rockham and Butterfield, 2s.; Two Birmingham Nurses, 2s.; Matron and Sister, Oxford Eye Hospital, 2s.; Stockton Nurse, 2s.; Nurses' Ward and Baines, Lincs., 2s.; V.A.D., 2s. ... ... ... ... ... ... ... 47 0

**Sums of 1s.:**

Nurse Sheppard, B.M.; A Member of College of Nursing; Sister Mary Coxeter; Nurse Cotton; A Nurse; Nurse Moxley; Nurse Jones; Q.V.N.I.; V.A.D. ... 9 0

**Total, 4,017 shillings.**

